# Herpes simplex keratitis – generalities and new perspectives

**DOI:** 10.22336/rjo.2026.49

**Published:** 2026

**Authors:** Camelia Constantin, Mihaela Elena Cernat

**Affiliations:** 1 “Dr. Carol Davila” Central Military Emergency University Hospital, Bucharest, Romania

**Keywords:** herpes simplex virus, keratitis, antivirals, ELISA = Enzyme-Linked Immunosorbent Assay, HEDS = Herpetic Eye Diseases Study, HSV = Herpes Simplex Virus, ICGA = Immunochromatographic Assay, IVCM = In Vivo Confocal Microscopy, MDH = Multiplex Dot Hybridization, PCR = Polymerase Chain Reaction, PK = Penetrating Keratoplasty

## Abstract

Herpes simplex virus (HSV) keratitis is a prevalent and potentially blinding condition driven by recurrent corneal infections. Globally, the disease affects approximately 1.5 million individuals annually, with 40,000 new cases progressing to severe visual impairment.

The distinct clinical manifestations of HSV keratitis require a comprehensive, layer-by-layer assessment of the cornea, encompassing the epithelium, stroma, and endothelium. Characteristically, active epithelial keratitis presents with classic dendritic lesions featuring terminal bulbs that stain vividly with fluorescein and rose Bengal. While typical presentations are readily identified, atypical cases necessitate advanced laboratory confirmation. Traditional diagnostic techniques include viral tissue cultures, enzyme-linked immunosorbent assays (ELISA), and cytological evaluation of Giemsa-stained corneal scrapings to detect pathognomonic multinucleated giant cells and intranuclear inclusions. Furthermore, modern modalities such as in vivo confocal microscopy (IVCM) and immunochromatographic assays (ICGA) are rapidly transitioning from research tools into promising point-of-care clinical adjuncts to enhance diagnostic precision.

Prompt and targeted therapeutic management is essential to avert permanent vision loss. Treatment strategies are strictly tailored to the affected corneal layer, utilizing specific combinations of topical or oral antivirals and corticosteroids. Beyond acute treatment, long-term antiviral prophylaxis plays a major role in suppressing viral reactivation and preventing disease recurrence.

Despite being an extensively researched pathology, HSV keratitis remains a leading cause of infectious corneal blindness worldwide. Staying focused on recent diagnostic and therapeutic advancements is imperative for clinicians to optimize medical protocols and achieve the best possible outcomes for every patient.

## Introduction

Herpes simplex keratitis is a common and potentially blinding condition caused by recurrent corneal infections with the herpes simplex virus (HSV). HSV is a common infection that primarily spreads by skin-to-skin contact and causes painful blisters or ulcers. Ocular involvement can present as a primary infection or recurrence from latent disease and can affect all ocular tissues. Although ocular HSV infection is usually due to HSV-1, which establishes latency in the trigeminal ganglion, HSV-2 can also cause HSV keratitis [1-3].

Globally, HSV keratitis incidence is 1.5 million yearly, including 40,000 new cases that result in severe visual impairment [4]. In addition to classical factors such as sunlight, trauma, fever, or heat exposure, COVID-19 infections and vaccinations are now considered risk factors for developing HSV keratitis [5,6].

## Disease

HSV keratitis is typically unilateral, though bilateral involvement may occur, particularly in patients with underlying immune dysregulation. The virus can affect all corneal layers through distinct pathophysiological mechanisms. For instance, active epithelial keratitis stems from direct viral infection and replication within corneal epithelial cells, whereas stromal keratitis is principally driven by immune-mediated responses [1]. Recurrent viral reactivations within the sensory ganglion induce progressive corneal scarring, tissue necrosis, and severe neurotrophic keratopathy.

## Clinical presentation

Patients typically present with blurred vision, photophobia, conjunctival redness, tearing, and severe ocular pain, which serves as a hallmark of acute epithelial involvement. Focal or diffuse reductions in corneal sensation can persist long after the infection resolves, with the severity and distribution of corneal hypoesthesia directly correlating with the extent, duration, and frequency of herpetic recurrences [7].

## Clinical examination

On slit-lamp biomicroscopy, early infection may present as coarse, punctate epithelial keratitis, which is easy to misdiagnose. As the disease progresses, it develops into pathognomonic, multiple arborizing dendritic epithelial ulcers that strictly follow the corneal nerve pattern and feature distinctive terminal bulbs (**Fig. 1**). Structurally, the ulcer bed stains vividly with fluorescein. In contrast, the swollen epithelial borders retain rose Bengal or lissamine green dye (**Fig. 2**). Notably, inappropriate topical corticosteroid use can cause these dendritic lesions to coalesce into extensive geographic ulcers. Associated clinical signs include mild conjunctival injection, ciliary flush, mild stromal edema, and subepithelial inflammatory cell infiltration. Following clinical resolution, a characteristic “ghost dendrite” often remains visible beneath the healed epithelial area (**Fig. 3**). Additionally, severe cases may be accompanied by blepharoconjunctivitis, resulting in lid lesions and follicular conjunctivitis [7].

**Fig. 1:**
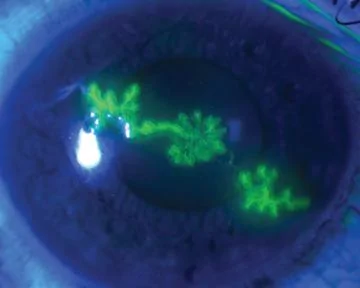
Fluorescein brightly stains the base of the HSV epithelial dendritic lesions (American Academy of Ophthalmology 2023-2024)

**Fig. 2:**
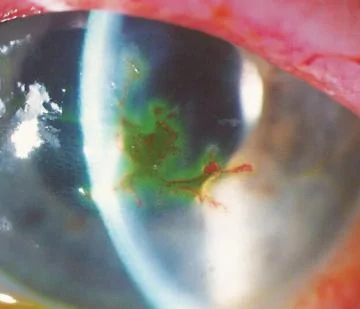
Combined fluorescein and rose bengal staining of geographic HSV keratitis (American Academy of Ophthalmology 2023-2024)

**Fig. 3:**
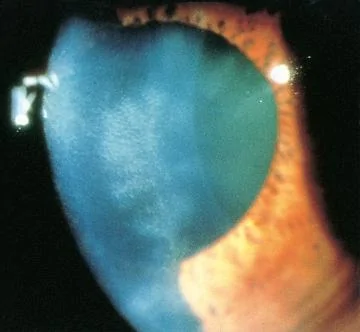
Residual stromal inflammation following dendritic epithelial keratitis may leave a ghost opacity or footprint of the dendrite (American Academy of Ophthalmology 2023-2024)

## Diagnosis

Establishing an accurate diagnosis requires differentiating HSV dendriform lesions from other ocular pathologies. Unlike the pseudodendrites observed in herpes zoster ophthalmicus, true HSV dendrites exhibit elevated edges and terminal bulbs. A comprehensive differential diagnosis must also exclude Acanthamoeba epithelial keratitis, adenoviral keratoconjunctivitis, Epstein-Barr virus keratitis, and simple epithelial deposits secondary to systemic diseases [8]. While clinicians primarily establish a definitive diagnosis clinically by identifying these structural corneal lesions, atypical or ambiguous presentations require advanced diagnostic confirmation through laboratory and molecular techniques.

To enhance diagnostic precision, modern high-resolution imaging techniques are increasingly integrated into ophthalmic practice. In vivo confocal microscopy (IVCM) offers non-invasive, layer-by-layer corneal analysis at a 1-μm resolution, which is critical for differentiating HSV from other microbial keratitis or corneal dystrophies. Pathognomonic IVCM features of active HSV-1 keratitis include epithelial microerosions, cellular distortion, squamous metaplasia, hyper-reflective subepithelial dendritic Langerhans cells, and marked keratocyte activation. IVCM enables the quantification of deep structural changes, including stromal fibrosis, endothelial keratic precipitates, and severe sub-basal nerve plexus degeneration. Because a profound reduction in sub-basal nerve density correlates with poor post-keratoplasty prognosis, IVCM has emerged as an essential tool for surgical decision-making and long-term postoperative monitoring [2,9].

When clinical signs are atypical, traditional cytological evaluation of Giemsa-stained corneal scrapings can reveal pathognomonic multinucleated giant cells and Cowdry A intranuclear inclusions. Conventional diagnostic modalities, including viral tissue culture and enzyme-linked immunosorbent assay (ELISA), also remain reliable adjuncts. However, molecular diagnostics have advanced substantially, offering rapid, point-of-care solutions. The office-based AmpliVue immunochromatographic assay (ICGA) provides rapid viral detection with a documented 84% sensitivity and 100% specificity. Similarly, the multiplex dot hybridization (MDH) assay utilizes targeted oligonucleotide probes to achieve high diagnostic accuracy, demonstrating 93.3% sensitivity and 100% specificity relative to real-time polymerase chain reaction (PCR). Furthermore, emerging diagnostic frameworks leverage detection of infected cell protein 0 (ICP0), an acute-phase protein that regulates viral expression, directly from the tear film. Combined with metagenomic deep sequencing, these advanced molecular platforms promise to significantly improve diagnostic accuracy and resistance profiling in complex or refractory clinical scenarios [9].

## Treatment

Antiviral treatment for epithelial keratitis is critical to shorten the clinical course and potentially reduce herpetic neuropathy. Therapeutic protocols vary based on geographic availability. According to the American Academy of Ophthalmology, in the United States, current options include ganciclovir ophthalmic gel 0.15% or topical trifluridine 1% drops administered eight times daily for 10 to 14 days, while acyclovir 3% ointment is unavailable. Globally, alternative regimens involve acyclovir ointment applied five times daily for 10 to 14 days, or systemic interventions such as oral acyclovir (400 mg five times daily) and oral valacyclovir (500 mg three times daily). Crucially, topical corticosteroids remain strictly contraindicated during active epithelial disease [7].

Managing stromal keratitis requires a balanced combination of anti-inflammatory and antiviral agents. Standard treatment involves prednisolone 1% drops administered three to six times daily, paired with either topical trifluridine four times daily, oral acyclovir (400 mg twice daily), or oral valacyclovir (500 mg two to three times daily). To minimize corneal toxicity during extended treatment courses longer than a few weeks, discontinue topical antivirals in favor of sustained oral antiviral therapy. All topical antiviral drugs are characterized by ocular surface toxicity, causing allergic reactions or punctal and nasolacrimal duct stenosis. Prolonged use of these formulations is not advised. Moreover, steroid medication must be withdrawn gradually, tapering the doses generally over a few weeks. The most concerning side effects of topical steroids include increased intraocular pressure, cataract, and secondary infections [1,8].

Evidence from the Herpetic Eye Diseases Study (HEDS) [10] confirms that co-administering topical corticosteroids with a prophylactic antiviral significantly shortens the duration of herpes simplex stromal keratitis. Furthermore, long-term suppressive therapy utilizing oral acyclovir reduces HSV keratitis recurrence rates. Prophylactic regimens typically consist of oral acyclovir (400 mg twice daily) or oral valacyclovir (500 mg once or twice daily), combined with fluorometholone 0.1% once daily. In cases that progress to necrotizing keratitis or geographic ulceration, management intensifies to prednisolone 1% drops every two hours, supplemented by systemic antivirals such as acyclovir (400 mg five times daily) or valacyclovir (500 mg three times daily).

The HEDS study on recurrence rate clearly demonstrated that short-course oral antivirals during active HSV epithelial keratitis do not prevent later stromal keratitis or iritis. In contrast, a 12-month course of prophylactic oral acyclovir (400 mg) twice daily significantly decreased the recurrence rate of stromal involvement. Although the HEDS study authors did not recommend a prolonged, beyond 12 months, acyclovir prophylaxis, clinical practice recommendations and observations seem to postulate a positive role of long-term prophylaxis, especially in patients with a high recurrence rate, significant corneal thinning at risk of corneal perforation, with comorbidities, such as atopy, autoimmune diseases, or in immunocompromised patients. Patients with a history of HSV keratitis undergoing surgical procedures, such as corneal transplant, photorefractive procedures, or cataract surgery, may benefit from acyclovir prophylaxis until the inflammation associated with the procedure and the risk of recurrence decrease.

Surgical intervention is indicated when severe stromal keratitis leads to structural complications, such as stromal perforation, or visually significant stromal scarring. For corneal perforations, acute management options include cyanoacrylate glue application, amniotic membrane transplant, or emergency penetrating keratoplasty (PK). When permanent stromal scarring severely impairs vision after HSV keratitis resolves, PK is the definitive treatment. The prognosis of PK remains favorable, with success rates ranging from 60% to 80%, provided the eye has remained free of active disease signs for at least six months before the procedure. Conversely, the risk of graft failure escalates significantly if PK is performed on actively inflamed eyes or in recipient corneas presenting with vascularization in more than two quadrants [7].

Looking forward, one future treatment strategy is to enhance patients’ immune resistance to infection through an HSV-1 vaccine. Currently, no vaccines are approved, but ongoing studies are underway. In a 2020 study, the authors identified 15 viral-encoded proteins as candidates for further testing in an HSV-1 vaccine [9].

## Conclusion

Despite being an extensively researched pathology, HSV keratitis remains a leading cause of infectious corneal blindness worldwide. Staying focused on recent diagnostic and therapeutic advancements is imperative for clinicians to optimize medical protocols and achieve the best possible outcomes for every patient.
